# Observation of Live Ticks (*Haemaphysalis flava*) by Scanning Electron Microscopy under High Vacuum Pressure

**DOI:** 10.1371/journal.pone.0032676

**Published:** 2012-03-14

**Authors:** Yasuhito Ishigaki, Yuka Nakamura, Yosaburo Oikawa, Yasuhiro Yano, Susumu Kuwabata, Hideaki Nakagawa, Naohisa Tomosugi, Tsutomu Takegami

**Affiliations:** 1 Medical Research Institute, Kanazawa Medical University, Uchinada-machi, Kahoku-gun, Japan; 2 Department of Medical Zoology, Kanazawa Medical University, Uchinada-machi, Kahoku-gun, Japan; 3 Department of Pathological Sciences, Faculty of Medical Sciences, University of Fukui, Matsuoka Fukui, Japan; 4 Department of Applied Chemistry, Graduate School of Engineering, Osaka University, Suita, Osaka, Japan; 5 Japan Science and Technology Agency (JST), Core Research for Evolutional Science and Technology (CREST), Kawaguchi, Saitama, Japan; Centro de Pesquisas René Rachou, Brazil

## Abstract

Scanning electron microscopes (SEM), which image sample surfaces by scanning with an electron beam, are widely used for steric observations of resting samples in basic and applied biology. Various conventional methods exist for SEM sample preparation. However, conventional SEM is not a good tool to observe living organisms because of the associated exposure to high vacuum pressure and electron beam radiation. Here we attempted SEM observations of live ticks. During 1.5×10^−3^ Pa vacuum pressure and electron beam irradiation with accelerated voltages (2–5 kV), many ticks remained alive and moved their legs. After 30-min observation, we removed the ticks from the SEM stage; they could walk actively under atmospheric pressure. When we tested 20 ticks (8 female adults and 12 nymphs), they survived for two days after SEM observation. These results indicate the resistance of ticks against SEM observation. Our second survival test showed that the electron beam, not vacuum conditions, results in tick death. Moreover, we describe the reaction of their legs to electron beam exposure. These findings open the new possibility of SEM observation of living organisms and showed the resistance of living ticks to vacuum condition in SEM. These data also indicate, for the first time, the usefulness of tick as a model system for biology under extreme condition.

## Introduction

Scanning electron microscopes (SEM), which image sample surfaces by scanning them with an electron beam, are widely used for steric observations of resting samples in various fields. Conventional SEM sample preparation methods comprise multiple procedures including fixation, conductive staining, dehydration, drying, ultrathin coating and mounting on a specimen stub. If a sample has low conductivity, it results in serious damage to the SEM images; hence, the sample is treated with conductive staining and metal or carbon coating. Conventional SEM is not a good tool to observe living organisms because of the associated exposure to high vacuum pressure and electron beam radiation.

SEM has been widely used since the 1970s to observe ticks and mites in acarology because it enables the observation of surface-fine structure. Various applications have been reported, and the usefulness has been widely shown in identification of new species, analysis of antiparasitic drugs, host-infection interaction, etc. [1]–[7]. Recent applications of low-temperature SEM analysis enabled observation of new morphological attributes and proposed a new model of leg locomotion [8], [9]. However, these observations did not involve living organisms or real movements. To obtain fine observations of legs and detailed movements of the capitulum, high resolution observation of living organisms is desirable. We hypothesized that if the living mites and ticks can be observed under SEM, we could record real-time videos of their actions. In addition, we could cross-breed mites and ticks and determine the species' characteristics more precisely if they survive after SEM observation. Until now, achieving this has been believed to be extremely difficult.

We accidently discovered that wild ticks were resistant to vacuum pressure. Ticks could walk inside desiccators connected to a vacuum pump (approximately 10 Pa). After 30 min, ticks were removed from the vacuum desiccator; most were alive under atmospheric pressure. On the other hand, it has been known that dead ticks without any conductive treatments were observable under SEM. These observations prompted us to observe living hard ticks using SEM under high vacuum conditions. Even under vacuum pressure (<1 Pa) and electron beam exposure, many ticks remained alive and moved their legs actively during and after the observation. To our knowledge, this is the first example demonstrating observation of living organisms using SEM in this field.

## Results

### Observation of *Haemaphysalis flava* (*H. Flava*) by light-microscopy

**Figure 1:**
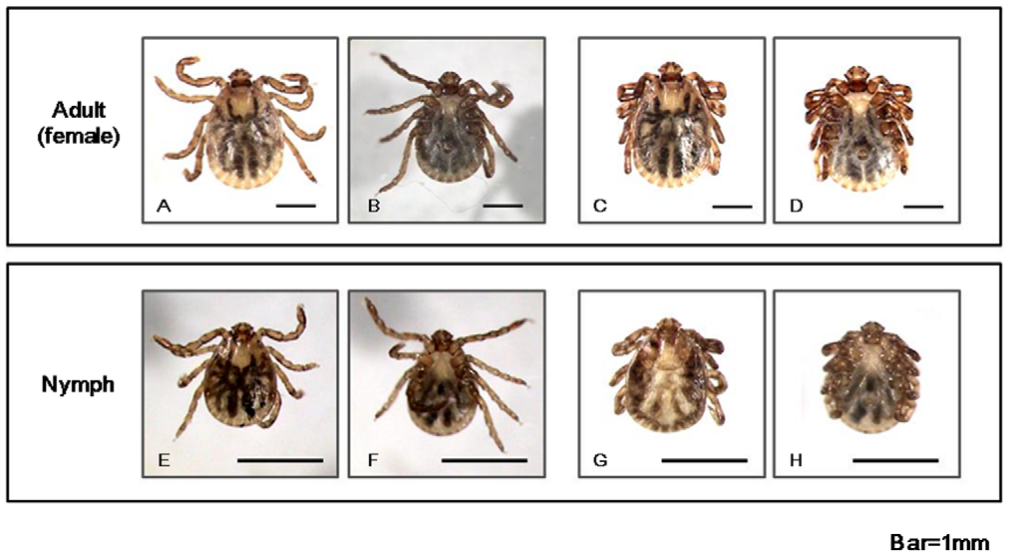
Photograph of adult and nymph *H. flava*. (A and B) Photograph of a living adult female. Back and abdomen are shown. (C and D) A dead adult female. (E and F) A living nymph. (G and H) A dead nymph.

*H. flava* was identified according to Yamaguti's protocol [10]. *H. flava* is a blood-sucking hard tick and is widely distributed in the Far East. Eyes are absent and the scutum is inornate. The second segment of palpi usually projects laterally beyond the basis capituli. Fig. 1 shows an adult female and nymph under a light microscope. Live ticks moved their legs continuously, but dead ticks had their legs tightly folded against their bodies.

### Observation of live ticks under SEM

**Figure 2:**
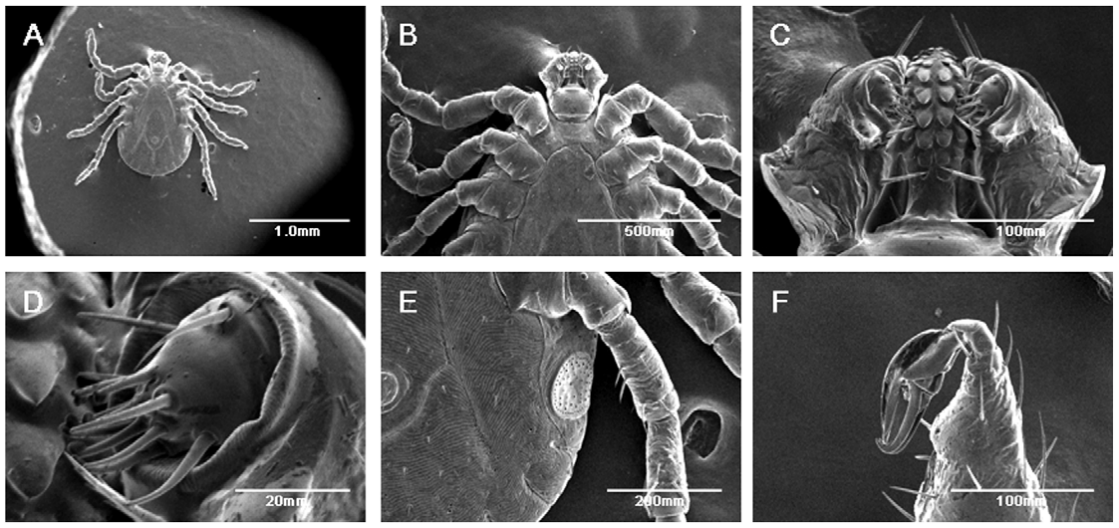
SEM observation of *H. flava*. (a) Whole body, (b) upper body, (c) capitulum, (d) 4^th^ article, (e) spiracular plate, (f) claw and pulvilus of nymph are shown.

We introduced a live nymph tick into Hitachi S3400N SEM. Clear images were obtained without any fixation or conductive treatment. We concluded that the surface of ticks sustained electron conductivity. Images were obtained without charge-up of the surface, and the body, eight legs, capitulum, hypostome, anus, spiracular plate, claw, etc. could be observed clearly (Fig. 2).

### Motion of ticks under SEM

**Figure 3:**
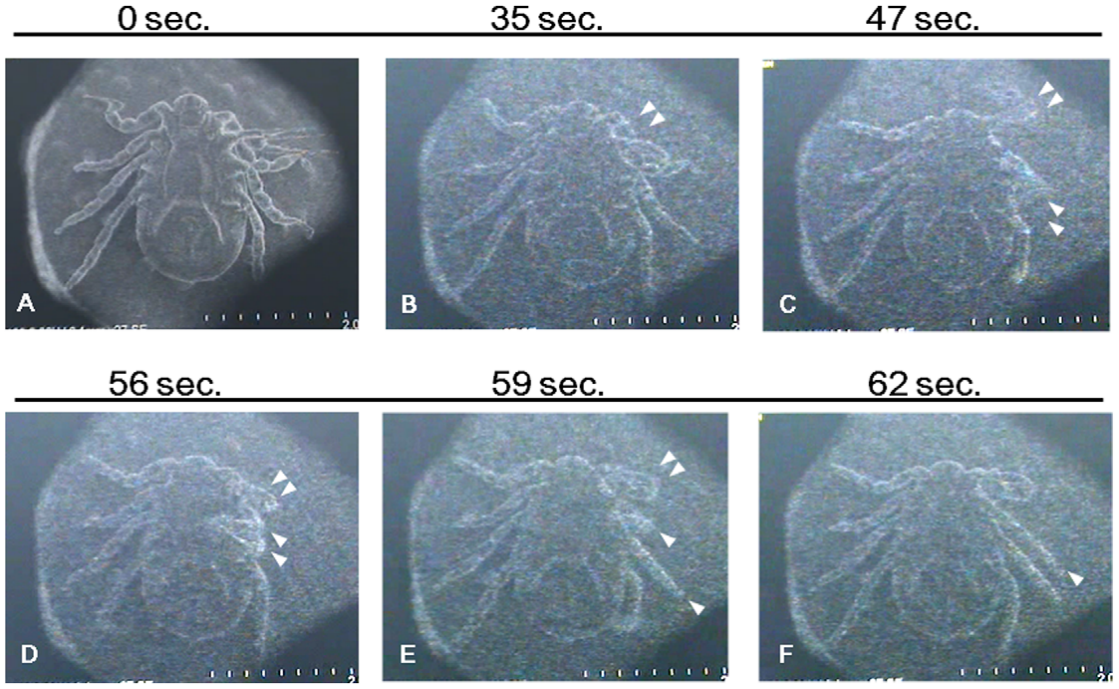
Motion of a live tick under SEM. Images of the TV mode were recorded. Time under vacuum conditions is shown on top of each picture. Leg movements are indicated by triangles.

Under the TV mode of SEM, we recorded a video of the SEM monitor after initiating decompression to 1.5×10^−3^ Pa and observed leg action. Fig. 3 shows representative pictures clipped from SEM monitor videos. The tick moved its second left leg actively for approximately one min. We observed this motion in both adults and nymphs. Ticks tested under SEM could move all four legs. High-resolution capture was possible, and the ticks could move their legs after the capture. Gradually, they stopped moving, but remained alive after being placed outside SEM (a walking tick on tweezers is shown in movie S1). The ticks were alive and could move after they were removed from the tape fixing them to the SEM stub. We repeated the observation with 20 ticks and obtained similar results. We also observed dead ticks under the same SEM conditions, but only a slight shrinkage of legs was observed with capture. We concluded that the action under SEM was the real motion of live ticks.

### Survival under SEM

**Figure 4:**
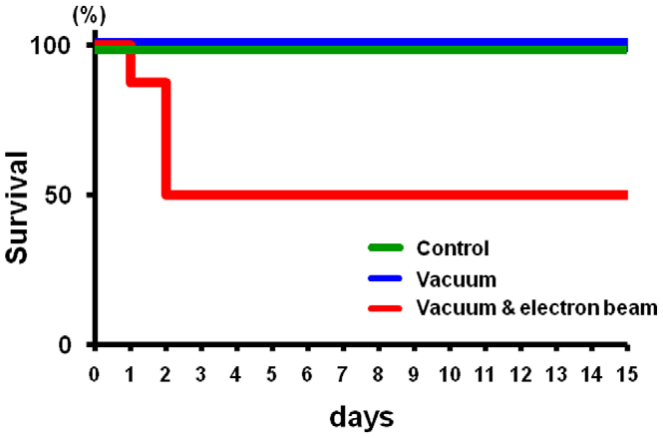
Survival of ticks after SEM observation. Nymph ticks were classified into three groups; exposed to vacuum with electron beam, exposed to vacuum and control with no treatment. Each group consists of eight ticks. % of survivors per tested group are shown.

We tested 20 ticks (8 female adults and 12 nymphs) with 30 minutes SEM observation and all of them survived for two days. Next, to determine the lethal effect of SEM observation for a longer period, we randomly classified nymph ticks into three groups (n = 8) - not exposed to vacuum/electron beam, exposed to vacuum with electron beam, exposed to only vacuum. Groups not exposed to the electron beam survived >2 weeks, whereas in the group exposed to the electron beam half of the ticks died within 2 days (Fig. 4). Thus, the electron beam but not vacuum is lethal to ticks. The ticks examined in the present study are characterized by the presence of spiracular plates connected to respiratory organ. We speculate that the ticks do not need any CO_2_-O_2_ gas exchange for 30 minutes but are sensitive to electron beam.

### Reaction of legs against capture mode

**Figure 5:**
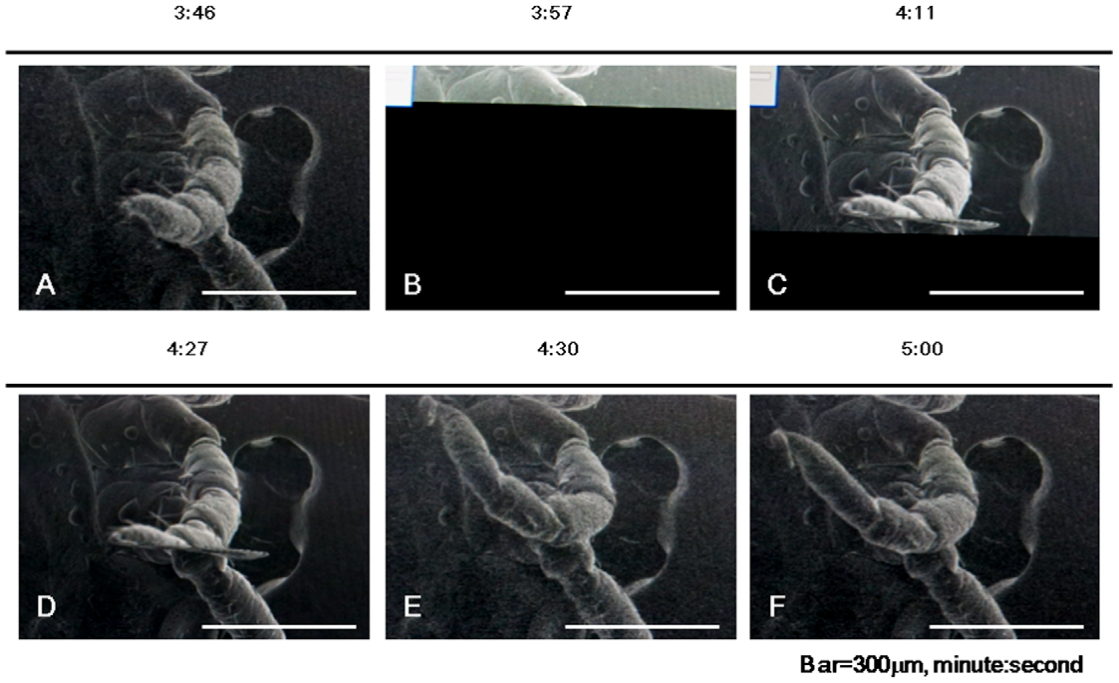
Example of response to scanning in capture mode. The continuous images of one tick are shown. Time under vacuum conditions is shown on top of each picture. (A) An image of TV mode before capture. (B and C) The scanned images. (D) A captured image. Distortion of image is clearly observed at the pulvilus. (E and F) The images of TV mode after D.

**Figure 6:**
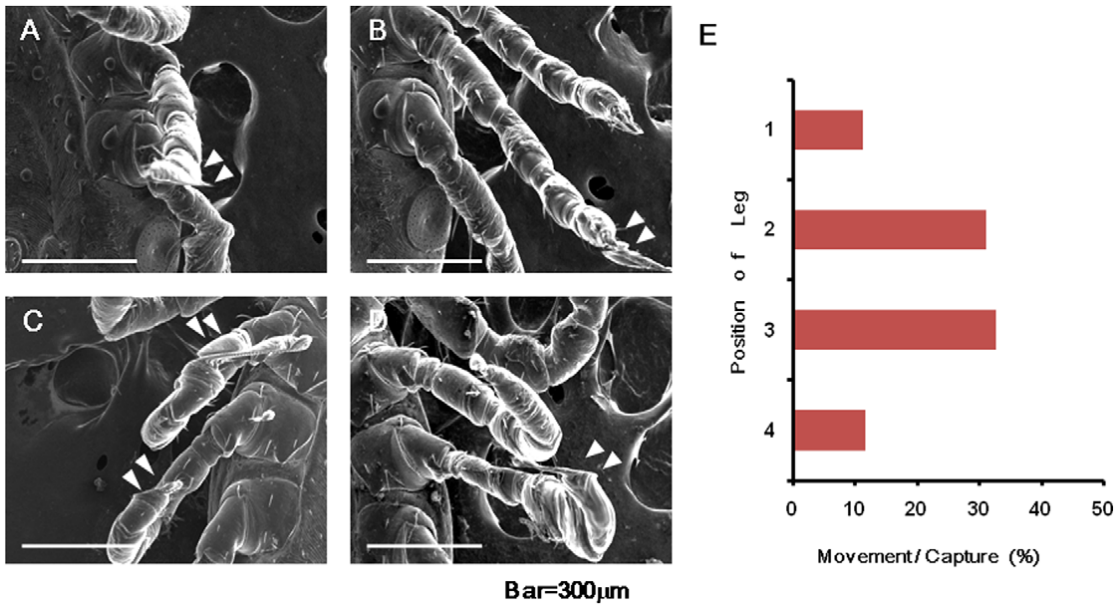
Movement of four different ticks during image capture. (A–D) An example of second leg and third leg. Distortion is indicated by triangles. (E) Frequency of movement from multiple experiments is shown. Totally, 178 captures were performed using 20 nymph ticks. Each leg was captured more than 2 times. The capture numbers were; 43 times for 1st leg, 46times for 2^nd^ leg, 45 times for 3^rd^ leg and 44 times for 4th leg.

When we recorded legs in capture mode, we noticed image distortion, possibly caused by a specific reflex movement of the ticks. Fig. 5 shows the representative captured images of one tick from videos. Ticks gradually cease their leg motion under SEM. However, during the capture mode, they moved their legs rapidly as if they could sense the scanning electron beam. The ticks moved when the beam reached the edge of the legs (around pulvillus) because the image was often distorted in that portion (Fig. 5 and Fig. 6A, B, C, D). We counted the frequency of this response and as shown in Fig. 6E. These results show that the ticks were alive during the SEM investigation and may further imply that they respond to irradiation by leg movement.

## Discussion

We performed real-time observation of live ticks using SEM. The ticks survived the SEM observation under vacuum condition, and we succeeded in recording their movement as a video (movie S1). The ticks were resistant to vacuum under our experimental condition (10^−3^ Pa for 30 minutes), but seemed to be sensitive to electron beam irradiation used to obtain the images (Fig. 4). Distortion of captured images in Figs. 5 and 6 seemed to reflect the tick's sensitivity to the electron beam.

The responses of mammals under experimental vacuum condition on Earth have been analyzed, and the results of accidental and experimental decompression have been reported in humans and monkeys [11]–[13]. It was concluded that the vacuum condition could affect behaviour and physiological condition and have lethal effect on mammals. Since vacuum systems developed, it has been widely believed that no animal can live in vacuum condition. However, the situation is different in lichens and bacteria. In space experiments, lichens and bacteria that had been exposed to space vacuum condition survived successfully [14]–[16]. In addition, it has been reported that tardigrades (trivial name is “water bear”) are resistant to vacuum condition in space and SEM [17], [18]. They were discovered in 18th century and belong to the invertebrate clade Ecdysozoa, which also comprises such animals as *Caenorhabditis elegans*. They are known to be resistant to various extreme environments reviewed in [19]. In recent experiments in Biopan-6 platform provided by European space agency, they were exposed to space vacuum under desiccated condition [17]. Surprisingly, they survived well after exposure to space vacuum alone, even though samples exposed to vacuum plus ultraviolet light-irradiation did not. However, they were resistant to extreme condition in the space under anhydrobiosis situation. Under this situation, they lose water from their bodies and become immobile. The change of DNA configuration and repair in the dehydrated Tardigrade cell is estimated as the mechanism of their tolerance but detailed mechanism has not been elucidated so far.

Different from tardigrades, *H. flava* in our experiments were hydrated and mobile. Then, the ticks keep their water inside of their body and condition is different from tardigrades in space. The ticks in the present study have a pair of spiracular plates, and they breathe through the stigma in them. Therefore, vacuum conditions may cause severe respiratory system damage and death. Actually, some anti-tick agents are emulsifying agents containing fatty acids such as sorbitan esters of fatty acids, which can seal the stigma [20], [21]. This implies that ticks can be choked to death. However, from our results, short-term choking did not have a lethal effect on ticks because they lived even under vacuum conditions in SEM for 30-min. Recently, the discontinuous regulation of breath was reported in insects [22]. Some insects quit breathing in order to avoid the damage caused by active oxygen. This result implied their resistance against hypoxic environment. We are speculating that ticks may display vacuum resistance if they have a similar breath-stopping function as shown in insects.

When leg images were captured with high energy electron beams, the ticks seemed to sense the electron beam. All responses took place at the edge of leg (Figs. 5 and 6). *H. flava* has a Haller's organ present only in the first legs, which can sense the surroundings [10]. Although the other legs also responded to the capture, no such organ was observed. We speculate two possibilities for the observed reaction in the legs: existence of an unknown sensor in legs and/or increased body surface temperature. However, we could not reveal the specific mechanism, and further study is therefore needed.

Recent advances in development of ionic liquids have enabled rapid and simple sample preparation for SEM observation of cultured human cells [23]–[25]. Ionic liquid is an alternative to the conventional coating treatment of SEM sample preparation. We applied hydrophilic and hydrophobic ionic liquids to ticks, but could not observe significant improvement in captured images as far as we tested. Some of them were very toxic to ticks and cause acute death (data not shown). Without coating, we obtained images of the body and coating layer, such as the cuticula, which may have provided conductivity to the body surface (Fig. 2). Therefore, the direct observation shown in the present study can be effective to some extent.

In conclusion, even under vacuum pressure (<1 Pa) and electron beam exposure in SEM, *H. flava* remained alive and moved their legs actively during and after the observation. Their real-time movements can be captured as SEM images. These results indicate that SEM enables more detailed visualization of tick motion. Further studies are required to test whether SEM observations are applicable to other species.

## Materials and Methods

### Ticks

Wild *Haemaphysalis flava* (Photographs are shown in Fig. 1) were collected in Kanazawa city, Japan in October, 2010. No specific permits were required for the described field studies. The place is a public facility and open to people. The location is not privately-owned or protected in anyway. Our studies did not involve endangered or protected species. Adults and nymphs were collected by flagging with a white felt flag. They were then kept in test tubes (TPP AG, Zollstrasse, Switzerland) with only water and leaves to maintain humidity. Totally 64 ticks (8 female adults and 56 nymphs) were used.

### SEM observation

Ticks were observed by stereomicroscopy (Olympus Corp., Tokyo, Japan), and recorded using a digital Handycam camera (Sony Corp., Tokyo, Japan). For SEM analysis, the dorsal side of ticks was softly pasted with stainless steel tweezers on an electrically conductive tape (Nisshin EM Corp., Tokyo, Japan), placed on a sample stub for SEM (Figure S1). Hitachi S3400N SEM (Hitachi High-Technologies Corp., Tokyo, Japan) was used for observations. Vacuum conditions included pressure of 1.5×10^−3^ Pa and accelerating voltage of 2.0–5.0 kV. Tick movement was recorded using the digital camera, and fine pictures were obtained using its controlling software. Screen of the SEM monitor was directly recorded using a CX4 digital camera (RICOH Company, Ltd., Tokyo, Japan).

### Survival test

After TV mode scanning under vacuum or vacuum only, each tick was transferred to a test tube and kept at room temperature. Their appearance was checked daily using the loupe. Live ticks could hold onto the wall of test tubes and walk. However, dead tick had fallen to the bottom of test tube, with their legs folded against their bodies.

### Response of legs

Ultra-high density image capture for legs was performed with 2.0-kV accelerating voltage at 35× magnification. Each leg was captured twice. Clear actions shown in distorted pictures were counted in 178 captures.

## Supporting Information

Figure S1 **Photograph of a tick on SEM stub.**
(TIF)

Movie S1 **Movie of a live tick in SEM.** This movie consists of three sections; sample preparation, motion of a tick in SEM and its living motion after SEM observation are shown.
(MPG)
