# Correction: Observation of Live Ticks (*Haemaphysalis flava*) by Scanning Electron Microscopy under High Vacuum Pressure

**DOI:** 10.1371/annotation/c81bc3a4-63e1-44a6-bf1e-f923087bfa71

**Published:** 2012-08-06

**Authors:** Yasuhito Ishigaki, Yuka Nakamura, Yosaburo Oikawa, Yasuhiro Yano, Susumu Kuwabata, Hideaki Nakagawa, Naohisa Tomosugi, Tsutomu Takegami

The current study focused on the study of living ticks, and reported their sensitivity to electron beam and leg response. However, the authors wish to acknowledge prior work that reported electron microscopy imaging of living insects, those reports should have been cited in the Introduction section and are listed below:

Pease RFW, Hayes TL, Camp AS, Amer NM (1966) Electron microscopy of living insects. Science 154:1185-1186.

Sokoloff A, Hayes TL, Pease RFW, Ackermann M (1967) Tribolium castaneum:

Morphology of "Aureate" revealed by the scanning electron microscope.

Science. 157 :443-445.

Hayes TL, Pease RFW, Camp AS (1967) Stereoscopic scanning electron microscopy of living Tribolium confusum. J Insect Physiol 13: 1143-1145.

Scharf D (1977) Magnifications: photography with the scanning electron microscope. New York City, New York: Schocken Books

In addition, we would like to correct the grant number reported in the article, the correct declaration for the funding information should be as follows:

'This research was funded by Kanazawa Medical University (S2010-2 and H2011-11). The funder had no role in study design, data collection and analysis, decision to publish, or preparation of the manuscript.'

Finally, the scales in Figure 2 are not correct. Scales shown in B-F are micro meter. 

