# Supplementary figures and images for: Observation of Live Ticks (Haemaphysalis flava) by Scanning Electron Microscopy under High Vacuum Pressure
Source: PLoS One. 2012 Mar 14;7(3):e32676. doi: 10.1371/journal.pone.0032676 (PMC3303806; doi:10.1371/journal.pone.0032676)

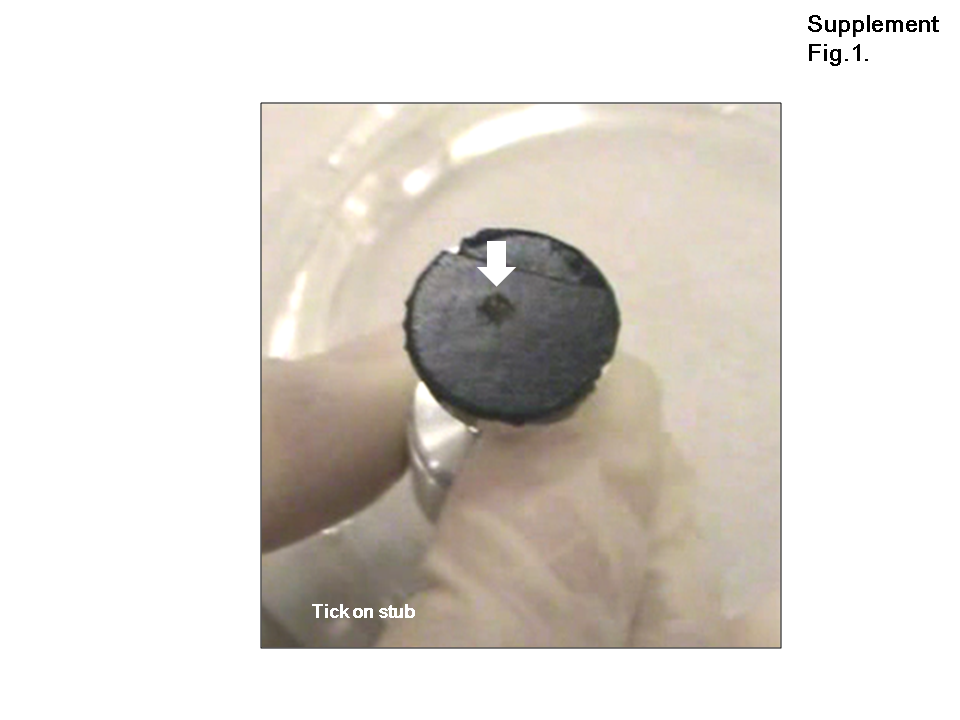

Supplement: Figure S1 — Photograph of a tick on SEM stub. (TIF) [file pone.0032676.s001.tif]
